# Health professionals' perspectives on clinical challenges in managing hypertensive disorders of pregnancy and recommendations for improving care: A multi-center qualitative study

**DOI:** 10.3389/fgwh.2022.968914

**Published:** 2022-11-10

**Authors:** Kwame Adu-Bonsaffoh, Evelyn Tamma, Adanna Uloaku Nwameme, Joyce L. Browne

**Affiliations:** ^1^ Julius Global Health, Julius Center for Health Sciences and Primary Care, University Medical Center Utrecht, Utrecht University, Utrecht, Netherlands; ^2^ Department of Obstetrics and Gynaecology, University of Ghana Medical School, Accra, Ghana; ^3^ Holy Care Specialist Hospital, Accra, Ghana; ^4^ Department of Social and Behavioural Sciences, School of Public Health, University of Ghana, Accra, Ghana

**Keywords:** hypertension in pregnancy, maternal hypertension, quality of care, challenges, health professionals, maternal mortality

## Abstract

**Background:**

Hypertensive disorders of pregnancy (HDP) are a leading cause of maternal mortality and morbidity globally despite the intensive international effort to improving maternal care. Substandard clinical care has emerged as a major contributing factor to the high maternal deaths associated with maternal hypertension globally and the impact is severer in low- and middle-income countries (LMICs). Context specific challenges impact negatively on the quality of maternity care and health providers play a crucial role in achieving positive pregnancy experiences for women. This study explored the perspectives of health professionals on the clinical challenges associated with the management of HDP in Ghana and recommendations for improving care.

**Methods:**

A multi-center qualitative study using in-depth interviews (IDIs) was conducted in five major hospitals in the Greater Accra Region of Ghana between June 2018 and March 2019. Health professionals (midwives/nurses and medical doctors) who have worked at the study sites for at least three months were included. Data were analysed based on thematic content using Nvivo software.

**Results:**

We included 62 health professionals comprising 40 midwives/nurses (64.5%) with a median age of 32.5 years (range 26 to 59) and 22 medical doctors (34.5%) with a median age of 34 years (range 25 to 55). Health providers highlighted major challenges associated with clinical management of hypertension in pregnancy: (1) patient-related factors (inadequate understanding and misconceptions about hypertension in pregnancy, women's non-compliance with clinical advice, financial constraints); (2) health system-related challenges (frequent unavailability of logistics, medications and laboratory support, delays in provision of care and limitations in the health insurance coverage); (3) health provider associated factors (inadequate number of health professionals and poor attitudes). Context-specific recommendations suggested for improving the quality of care in managing maternal hypertension include restructuring of the health system to reduce delays in providing care, improving financial coverage of medical insurance, encouraging social/family support, enhancing education on HDP and strengthening the health workers’ numbers and working conditions.

**Conclusion:**

Major challenges in the clinical management of HDP relate to the health system, health professionals and pregnant women themselves. Context-specific interventions are required to improve the quality of care for hypertensive mothers including regular health education, re-structuring of the health systems, refresher courses for health providers, improvement in health insurance coverage and government subsidy for hypertensive women.

## Introduction

Globally, maternal mortality and severe morbidity continue to present a major clinical and public health challenge to efforts aimed at achieving the Sustainable Development Goal (SDG) 3, to ensure healthy lives and promote well-being for all ages (1-3). The SDG's maternal health target aims to reduce the global maternal mortality ratio to less than 70 per 100,000 live births by 2030 (3). Unfortunately, there remains a wide disparity in the distribution of maternal mortality with unacceptably high rates in low- and middle-income countries (LMICs) (4, 5). For instance, the maternal mortality ratio estimates for Western Europe and Sub-Saharan Africa are 5 and 533 per 100,000 live births respectively indicating unacceptably high inequity (6).

The major causes for maternal deaths have been extensively studied and include obstetric haemorrhage, maternal hypertension or hypertensive disorders in pregnancy (HDP), sepsis and abortion related complications (4). While many local and international interventions have been instituted and significant progress achieved in terms of coverage of maternal health interventions and services, the progress to eliminate preventable maternal deaths has been slow. Hypertensive disorders including preeclampsia are the most common medical condition in pregnancy and are associated with worse pregnancy outcomes. In LMICs, preeclampsia accounts for about 15% maternal deaths with associated severe maternal and perinatal morbidities (5, 7, 8).

In Ghana, there is nearly 100% coverage for antenatal care for pregnant women with about 80% facility-based delivery rates (9). However, the maternal mortality in the country remains disproportionately high with a life-time risk of 1 in 82 compared to 1 in 11,700 in Western Europe (6). Although haemorrhage and maternal hypertension are the two leading causes of maternal mortality in the country, the relative proportion of the latter has doubled over the past decade with significant reduction in the former (9). Current evidence indicates that hypertensive disorders in pregnancy are the leading cause of institutional-based maternal deaths in Ghana (10, 11) and other countries in SSA have reported similar findings (12, 13).

Evidence-based interventions including locally appropriate measures are needed to further improve the quality of care for women with maternal hypertension. More recently, substandard clinical care has emerged as a major contributing factor to high maternal deaths associated with maternal hypertension globally although the impact is more severe in LMICs (14, 15). To optimally reduce the maternal deaths and severe morbidity attributed to hypertension in pregnancy there is the urgent need to actively involve all the relevant stakeholders in the decision including the healthcare providers. The perspectives of the doctors and midwives/nurses on the clinical care for hypertensive mothers are indispensable in identifying the barriers and facilitators to improving the quality of maternal care in the country. The objective of this multi-center qualitative study was to explore healthcare providers' perspectives on the challenges associated with the provision of maternal care for women with hypertension in pregnancy. The clinical experience of the healthcare providers would generate comprehensive evidence-based measures to improve the quality of care for pregnant women with maternal hypertension.

## Materials and methods

This multi-center qualitative study was conducted in five major hospitals in the southern zone of Ghana namely: Greater Accra Regional Hospital, La General Hospital, Kore-Bu Teaching Hospital (KBTH), Tema General Hospital and Lekma Hospital. All the five health facilities are located in the Greater Accra Metropolitan Area (GAMA) with a population of approximately 4 million. In the GAMA, there is about 98% of antenatal care coverage by skilled providers with a facility-based childbirth rate of about 92%, with most deliveries undertaken in public hospitals (9). The included health facilities conduct between 5,000 to 11,000 childbirths annually and cesarean section rates are between 30 and 45%.

This qualitative study is part of a larger study [Severe Pre-eclampsia adverse Outcome Triage (SPOT) study]. The main aim of the SPOT study was to externally validate risk prediction models for adverse maternal and perinatal outcomes in severe preeclampsia (16, 17). The detailed methodology of the SPOT study has been pulished elsewhere (18). The qualitative component assessed the quality of care associated with the clinical management of maternal hypertension based on the perspectives of the health providers and affected patients. In this paper, we report the clinical challenges associated with the care for women with HDP based on the perspectives of the health providers. The perspectives and lived experiences of care by the affected hypertensive mothers have been explored in a forthcoming paper (19).

Inclusion criteria comprised maternity health workers (doctors and midwives/nurses) who had worked at their current facility for at least three months, and provided written informed consent. We excluded health professionals who have worked in the health facility for less than three months.

### Participant recruitment and interviews

We recruited the study participants *via* purposive sampling and in-depth interviews (IDI) were conducted. In each study site, potential health professionals were approached individually, and the study protocol explained to them. Study identification number was assigned to those who agreed to participate in the study and a specific date was scheduled for the interview. All the study participants provided written informed consent prior to the interviews (IDIs) which commenced from 1 June 2018 and ended on 31 March 2019. The IDIs continued till saturation point was reached when subsequent interviews yielded no new themes. Saturation in data collection for qualitative research describes the point where no additional or new information is generated indicating the endline to stop collecting further data (20, 21). At saturation, the researcher obtains similar or same responses over and over again with further interviews. In this study, all the in-depth interviews (IDIs) conducted were audio-recorded. A semi-structured interview guide was used for the IDIs which were all conducted in English. A special room was specifically dedicated for the interviews in each health facility to prevent frequent disruptions during the course of conducting the IDIs. The duration of each IDI was between 30 and 45 min.

### Data management and analysis

Data were analysed based on the thematic content using the inductive analytic framework approach. The derivation of the themes were data-driven with minimal influence from the theoretical background (22). Transcription of the interviews commenced alongside the data collection and continued till the point of saturation. Initially, a codebook was developed to facilitate the thematic content analysis. To familiarize ourselves with the data, the transcripts were read severally in a more recursive manner by two members of the research team to identify the commonly occurring themes. This recursive reading of the transcripts was necessary to appreciate and understand the perspectives or the worldview of the study respondents. The notes taken during the interviews contributed significantly towards identifying the thematic areas that emerged from the transcripts. This resulted in the creation of the initial codes from the transcripts which were crucial to the final coding. The coding was undertaken by two members of the research team and inconsistencies were discussed between them. In case of any unresolved disagreement, further discussions were held with the other research members. The codes and the emerged themes were discussed and agreed on among the members of the research team. NVivo software (version 12) was used for the coding and analysis. We ensured adequate triangulation of the results by including healthcare providers of different categories (medical doctors and midwives of different clinical experiences) from different health facilities (data source triangulation) (23).

## Results

In this qualitative study, 70 health maternity care professionals were eligible, out of which 62 participated comprising 40 (64.5%) midwives/nurses and 22 (35.5%) medical doctors. The median ages of the midwives/nurses and medical doctors were 32.5 years (range 26–59) and 34 years (range 25–55) respectively. Table 1 shows the socio-demographic characteristics of the participants. The responses from the participants highlighted the important contemporary issues in the clinical management of hypertension in pregnancy in Ghana. The prominent themes that emerged from the narratives concerning the clinical problems in the management hypertension in pregnancy included patient-related challenges, health system-related challenges and health provider level challenges.

**Table 1:** Characteristics of study participants.

Variable	Total (*N* = 62)	Percentage (%)
Age categories (years)		
25–30	16	25.8
31–40	32	51.6
41–50	8	12.9
51–60	6	9.7
Position		
Midwives/nurses	40	64.5
Doctors	22	35.5
Gender		
Female	45	72.6
Male	17	27.4
Length of stay with facility		
<1 year	6	9.7
1–5 years	27	43.6
6–10 years	17	27.4
11–20 years	9	14.5
≥21 years	3	4.8
Hospital		
Korle-Bu Teaching Hospital	16 (6 doctors, 10 midwives/nurses)	25.8
La-General Hospital	14 (4 doctors, 10 midwives/nurses)	22.6
Lekma Hospital	9 (4 doctors, 5 midwives/nurses)	14.5
Greater Accra Regional Hospital	14 (4 doctors, 10 midwives/nurses)	22.6
Tema General Hospital	9 (4 doctors, 5 midwives/nurses)	14.5

### Theme 1: patient-related challenges

Challenges related to pregnant women themselves may hinder the provision of optimal maternal care. The health providers described client-related factors associated with sub-optimal quality of care for women with hypertension in pregnancy. These include inadequate women's knowledge on hypertension in pregnancy, spiritual beliefs and misconceptions, non-compliance with medical instructions, financial constraints and inadequate family support.

#### Women's inadequate knowledge on hypertension in pregnancy

A very common running theme recounted by the health professionals was inadequate knowledge on hypertension in pregnancy and preeclampsia displayed by the women. Lack of basic understanding about maternal hypertension and its complications presents a major challenge in the clinical management as patients frequently do not understand or appreciate the need for hospital admission especially in severe cases.

*“Majority of them [pregnant women] do not understand it because they’ve never had hypertension before, they’ve never been told they have hypertension and you [the woman] just come and we tell you, ‘You have hypertension’. Some of them do not understand what it is.”* (Resident doctor, 31 years)

“*In my opinion, some of the challenges are that most of these women have very low educational background so they might not really understand the severity of their condition.”* (Specialist doctor, 32 years)

The health workers emphasized the extent of pregnant women's ignorance and disbelief about HDP and potential for adverse outcomes. One midwife remarked that some of the women do not know the potential dangers associated with hypertension in pregnancy as well as and harbour disbelief about having hypertension.

“*Ignorance because she has no idea this [hypertension in pregnancy] is a dangerous disease. Once she comes to the clinic… once she is told she has hypertension she goes home, doesn’t come back again or she didn’t even believe what you told her so she goes she doesn’t come back again.”* (Midwife 40 years)

Relatedly, almost all the doctors and midwives/nurses overwhelmingly reported that health education promotion on hypertension in pregnancy are either non-existent or extremely minimal. They believed that the low knowledge and awareness levels of preeclampsia exhibited by the pregnant women are mostly due to lack of education on the subject. Some people stated that they hardly hear health promotion programs on hypertension in pregnancy *via* the media compared to the other medical conditions like malaria.

“*The education is not enough, we need to do more than what we are doing now. I hardly hear someone talk about hypertension in pregnancy on radio. The last time I heard was a lady who had a foundation, and she lost her baby … I had friends who were like ‘Is there something like that? [hypertension in pregnancy]’. But you will hear[about] the others [diseases] like malaria, but you will hardly hear people talk about hypertension in pregnancy.”* (Midwife, 40 years)

#### Spiritual beliefs and misconception

Deep-rooted spiritual beliefs and misconceptions were considered major factors contributing to substandard care. Most of the health providers stated that one major obstacle they encounter frequently is the issue of “dominance and control” of pregnant women by spiritual leaders such as pastors and herbalists. Most women had a lot of misconceptions concerning the causes of hypertension in pregnancy, with significant attribution to spirituality. The health professionals hinted that majority of women usually seek help from the religious leaders who “keep them” and only make referrals to hospitals when severe complications occur. Such delays in reporting to the medical facilities constitute a major challenge in managing HDP, as early detection and treatment are key.

“*You see [in] our part of the world we tend to be more into religion. Sometimes some of these women might have stayed in ‘prayer camps’ and other places for a long time and when things get out of hand then they come to the hospital to seek care which sometimes might be too late for them.”* (Specialist Obstetrician, 32 years)

However, the health professionals continue to educate the mothers to appreciate that preeclampsia is a medical condition and not spiritually related. In some cases, the hypertensive mothers request for prayers from their spiritual leaders before they consent to medical treatment. Spiritual or religious inclinations and misconceptions contribute significantly to delay in initiation of treatment and may be associated with worse adverse outcomes.

“*Even when they come for admission we go ahead to sometimes tell them that once you are here this is the condition [and] it is not spiritual. If you still believe it just add your prayers to the fact that the doctors are also doing their best for you. That is how we go about those things but some of them will still insist (..) their pastor (..) agrees.”* (Midwife, 30 years)

The need for regular and coordinated client education was mentioned as an important intervention to improving women's understanding of hypertension in pregnancy. The health providers recommended that the health education should not be restricted to the hospital setting but the whole society should be involved by means of social media campaigns.

“*The education should be done very well not in only the hospital; we should go out, the radio, the community; we should go and talk to them about the condition, how serious it is and why they should always see a doctor in case they have any problem like that.”* (Medical Officer, 38 years)

The providers believed that optimal knowledge about hypertension in pregnancy and its consequences will have positive impact on preventing major complications and improving clinical management. In some facilities, the introduction of maternal class into antenatal care is an important intervention to improve the health education promotion. However, the maternal classes are not organized frequently and not in all the facilities.

“*They have a maternal class that goes on every other week on Saturdays. That’s where they address almost all those issues: diabetes in pregnancy, hypertension in pregnancy and all those issues including breast feeding and everything.”* (Midwife, 30 years)

#### Non-compliance with medical advice

The doctors and midwives recounted several instances of misunderstanding between the health providers and patients during the course of providing care. Most of these conflicts are associated with patients' noncompliance with the prescribed treatment or refusal of hospital admission recommended by the health workers. In some situations, the patients present with hypertensive emergencies that require urgent initiation of treatment but some of them refuse admission and prefer to seek advice from their spiritual leaders.

“*Somebody comes in with imminent eclampsia, you want to admit and they are telling you they want to go and talk to their pastors first before they will agree to any interventions, so occasionally we get such situations or misunderstanding.”* (Medical Officer, 37 years)

#### Financial constraints

An important theme that emerged repeatedly from all the study sites was the chronic financial constraints experienced by the women with hypertension and their families. There were several instances where women could not afford the bill for their prescribed medications. Generally, these women require additional drugs to control the preeclampsia compared to the general normotensive pregnant women populations.

“*Most of these patients, their main problem is the money to buy their medications. They always complain of the medications being expensive or they don’t have money to buy, to do the lab [tests] because it's too expensive.”* (Medical doctor, 32 years)

Similarly, women with preeclampsia undertake several laboratory tests to guide the clinical decision by the health professionals. Multiple laboratory tests are required to assess the severity and integrity of the various organs that could be affected by the disease. In instances where the women are unable to pay for laboratory services, the health professionals are left in limbo as they do not have the laboratory support necessary to guide the clinical management.

#### Family support system and education

Family support system was a prominent theme that emerged from almost all the categories of health professionals. It became apparent that the clinical management of women with hypertension in pregnancy is multi-dimensional and the role of the family members is indispensable in achieving treatment success and preventing major complications. Adequate health education promotion for the immediate family members will improve their understanding and facilitate co-operation and social support.

“*For family members, I think it's more of education. Public Health educating the masses so they know the dangers associated with hypertensive disease and once they have any pregnant woman in their midst they should keep an eye on such women- those who may have hypertension along the line in their pregnancy.”* (Medical doctor, 37 years)

Another important consideration that emerged repeatedly was the need for the close family members including the partner to accompany the pregnant women to the health facilities for antenatal care services. Active involvement of family members in the antenatal care will provide the opportunity to learn about preeclampsia and other conditions in pregnancy which can improve the quality of care for the women especially at the home settings.

“*Family members, especially the partners, they should come with their wives to antenatal. As we explain things to their wives, their husbands pick it up. They will help manage the case at home because it's not all hypertensive disorders in pregnancy that are on admission. Majority of them are also on ‘admission’ at home and we will need vigilance there too and their partners can help in that.”* (Medical doctor, 26 years)

### Theme 2: Health system-related challenges

Majority of health professionals cited health system challenges as a major issue that hampered the quality of care for women with hypertension in pregnancy. These health system related challenges include irregular supply of medications, logistics and instruments for monitoring patients.

#### Shortages in medications and monitoring devices

Most of the interviewees reported that medications for treating severe hypertension were frequently unavailable. The health workers remarked that the frequent shortage of essential antihypertensives accounts for significant morbidities associated with maternal hypertension in the country.

“*The drugs are not always available. Sometimes, you write it in the folder, it's not there; patient has to buy it.”* (Midwife, 43 years)

Also, majority of the health professionals were emphatic on the issue of inadequate blood pressure measuring devices in most of the maternity units. Almost everyone cited the problem of frequent breakdown of the instruments due to high patient load. In addition, the challenges with the repair of these non-functioning instruments were frequently mentioned. The frequent shortage of the blood pressure measuring instruments frequently leads to sub-optimal monitoring of women with severe preeclampsia and this might be associated with poorer pregnancy outcomes. The high patient turn-out with overuse and the limited supply appeared as some of the underlying reasons for the situation:

“*You need a BP apparatus to work with, you go to the stores, there is none. Sometimes two rooms will have to share one.”* (Midwife, 40 years)

“*BP apparatuses, because of the rate at which we use them it gets spoilt quickly and we don’t have enough, at least we have to get three or four at the ward.”* (Midwife, 36 years)

“*And for the logistics, the instruments and gadgets; the BP machines, some are not in good working conditions … Because of the pressures on the ward … [BP machines] are not durable enough: just one BP machine taking care of 50 patients, so within months it's spoilt and getting one becomes a problem. So sometimes you have to go to another ward to borrow a BP machine … and use [it] on the ward. All these are creating problems.”* (Midwife 30 years)

An ultrasound scan for the initial assessment of the pregnancy is problematic in most cases. Some doctors complained about the unavailability of ultrasound scan during the 24-hour duty. Some participants deemed the ultrasound scan very necessary to confirm or monitor the fetal wellbeing especially in emergency situations during the night shift.

*“At 2 o’clock [p.m.] the ultrasound people will tell you they have closed, so it becomes difficult if a woman comes and you urgently need an umbilical artery doppler. Such women when they are in labour you need to closely monitor them on CTG, and we don’t have CTG papers so we are monitoring on screen we can’t print anything and we can’t reproduce the result to a specialist or someone.”* (Medical doctor, 28 years)

#### Laboratory challenges

Both the doctors and midwives shared their experiences related to the challenges associated with the laboratory tests performed for women with hypertension in pregnancy. The main concern was the delay in obtaining the results of the various tests performed because it usually takes days before the results arrive. The laboratory reports are vital for clinical decisions on a daily basis.

“*Laboratory support is another major challenge. They have to be doing all those labs continuously and then they don’t have the finances for that.”* (Senior staff midwife, 30 years)

“*Some of the ‘labs’ we request for women with hypertensive diseases, at a point we couldn’t get them done here so the samples had to be taken outside and you get them in 2–3 days and it makes decision making difficult.”* (Medical doctor, 28 years)

Some doctors complained about the fact that the laboratory unit does not work at night making their work very difficult. They sometimes get frustrated especially when laboratory support is needed urgently to supplement the treatment or diagnose complications of hypertension in pregnancy.

“*In this hospital, my only challenge with the ‘lab’ will be the nights. They don’t come at night so if you have a case at night they will have to go to an outside lab- that's frustrating sometimes.”* (Medical doctor, 28 years)

#### Challenges with blood products

Blood and blood products are important in the clinical management of preeclampsia and other hypertensive disorders in pregnancy. Majority of doctors and nurses recounted their unpleasant experiences with scarcity of blood products for obstetric patients especially in emergency situations. Frequent reports of unavailability of these essential treatment adjuncts hinders the treatment of severe preeclampsia especially when it is complicated with coagulopathy.

“*Sometimes you need blood urgently and they tell you there is no blood.”* (Medical doctor, 32 years)

“*It depends on the blood type and its availability at our blood banks. So as long as the blood is available at the bank they will issue it. Unfortunately, sometimes at the blood bank [they can’t] …, because it's serving so many patients.”* (Medical doctor, 42 year)

Sometimes it becomes difficult to secure blood for the women especially in emergency situations due to shortage of the blood products. In some situations in which blood is urgently needed, relatives of patients are sent to search for blood and blood products from other health facilities. Such frequent shortage of blood products especially in emergency cases puts the patients at risk of severe morbidity and might result in maternal death. In most of the health facilities, relatives or partners of pregnant women are requested to donate one or two units of blood to save for emergencies cases. Most of the relatives or partners are prepared to donate blood when their family member urgently needs it.

“*So what happens is that we have to take samples from the patient's blood, fill the blood bank form and then we give it to the patient's relative to take it to ‘37’ or Korle-Bu to donate and bring the blood back.”* (Medical doctor, 38 years)

“*The availability of blood and blood products is an issue. There are instances where we have to close the ER [Emergency Room] because of the non-availability of blood and we do case selection in terms of management. We have to liaise with other hospitals for blood and blood products.”* (Medical doctor, 31 years)

“*Usually, they are willing to donate if they have the emergencies and their relatives need it then they can see they need blood. That's when you see them rushing to donate.”* (Medical doctor, 31 years)

#### Unavailability of bed space

Another important finding frequently mentioned in all health facilities was recurrent shortages of beds for admitting pregnant women with obstetric emergencies, commonly referred to us as “no bed syndrome”. This normally occurs when the patient load is high and all the beds in the health facility are occupied. In most cases, pregnant women are referred to other hospitals if there are no beds at the time of reporting to the emergency room (ER). Some doctors stated that referring such patients to other hospitals is necessary to prevent severe complications which could arise as the patient is waiting to get a bed space. Usually, doctors triage the emergency cases and attend to the more severe ones first when the patient load is high.

“*Once there is no bed you don’t expect a pregnant woman to come and sit on the floor and deliver; you don’t expect one in early labour to wait as she might need caesarean section or else might rupture [uterine rupture]. If the place is choked, we refer to other hospitals.”* (Medical doctor, 31 years)

“*Sometimes when the whole place is full, emergency is full, theatres choked and all that we have lined up are emergencies and we have to choose among the emergencies again. So then when the case comes we triage and say with this one [patient] we can put this intervention in place.”* (Medical doctor, 31 years)

Severe obstetric cases usually require critical monitoring and treatment at the intensive care unit to optimize the needed care. Most of the health workers frequently described the reality of non-existence of ICU in the health facilities for critically ill obstetric clients. Unavailability of intensive care units (ICU) hamper optimal management of complicated cases of HDP which require strict monitoring and can potentially contribute to maternal mortality. The ICU is considered instrumental in the management of severe obstetric cases for continuous monitoring and lack of such facilities constitutes a major limitation in maternal care provision.

“*We try our best to stabilize the patients, but such people need ICU care and we don’t have ICU care here. They need continuous monitoring. Some might need ventilators; some might need dialysis and we don’t have such facilities here so we normally refer such cases.”* (Medical doctor, 31 years)

#### Challenges with health information management and communication

Inaccessible medical information was a major running domain in the narratives of the health professionals. In most cases, the previous medical records of the women are not obtainable due to poor health information storage in the health facilities and lack of electronic data management systems. Access to previous medical records is key to early diagnosis and initiation and optimization of treatment. However, medical information documentation and storage remains a real-time challenge as pointed out vividly by one of the participants.

“*What you will find out is that they will tell you she delivered in this hospital. You go and request for her folder to see what was recorded for past pregnancy and the folder is not there. They will end up giving her a new folder and you will always see her as a new client although she has a history.”* (Medical doctor, 26 years)

Poor communication between referring and referral facilities was also considered a major challenge. The health professionals in the referral center are not aware of or are not given adequate information about the emergency case being transferred. Lack of prior inter-facility communication about the referred cases and inadequate accompanying medical information might result in suboptimal care at the treatment center. On the other hand, some of the referrals were wrongly referred to the tertiary centers as they could have been managed at the secondary level facilities.

“*So you are just here and a referral comes and you are not prepared for the referral. You normally have to move from a health center, you go through a clinic, to a hospital, to the regional hospital before you get to a teaching hospital but a clinic can just refer straight and by pass all of that to a teaching hospital. We get referrals which do not need to come to Korle-Bu.”* (Medical doctor, 32 years)

#### Delay at the health facility

Delay in providing care at the health facility is a major factor associated with adverse pregnancy outcomes. Some of the health professionals pointed out that institutional delays in provision of appropriate treatment could result in complications. Typical examples of facility-based delays include wrong or late diagnosis and failure to initiate appropriate treatment.

“*In terms of the causes of the complications, there are certain delays and one of the delays is the delay that happens in the hospital facility be it in the referral centre or the teaching hospital.”* (Medical doctor, 31 years)

Similarly, some patients are kept in a queue for a long time prior to clinical consultation. It was pointed out that some patients wait for a long time before they are attended to by a doctor. On the other hand, the patients do not usually wait for long periods prior to medical consultations with the midwives.

“*At the antenatal clinic there are instances that the patients show up in the morning around 7, 8 and they leave, especially if they have to see a doctor, they may leave around 2, 3 o’clock. It can take hours but if they are not seeing a doctor but only a midwife then just after the consult with the midwife they can leave.”* (Medical doctor, 37 years)

#### Challenges with national health insurance scheme

The health professionals frequently mentioned some intrinsic limitations in the existing national health insurance scheme (NHIS) which hinder provision of optimal care for hypertensive mothers. Most of the health professionals indicated that the NHIS does not cover all the hospital bills for the hypertensive mothers. There was a general impression that the NHIS covers only part of the antihypertensive medication and the laboratory tests which are generally costly.

“*NHIS covers for hypertensive drugs but then there are certain categories of medication that are beyond the cover of the NHIS and for those ones we write for the patient but generally the normal ones are covered.”* (Medical doctor, 31 years)

“*It is expensive because even though NHIS covers for some of the drugs, the labs, not all are covered; and sometimes these people, even before they deliver, might have been on admission for a long time.”* (Medical doctor, 32 years)

Another related challenge that emerged from the interviews was that some of the women were not registered under the NHIS and this made their hospital admission extremely costly. These are usually women of low socio-economic class.

“*One other issue too is that there are instances where you admit patients, the Bp drugs are available they may not have the insurance and they may not have money to get it. So sometimes it becomes a challenge especially the emergency medications.”* (Medical doctor, 37 years)

### Theme 3: Health professionals-related challenges

#### Inadequate number of health professionals

Majority of the doctors and nurses complained about the mismatch between the high patient load and the inadequate number of health professional. Sometimes, the high patients load precludes optimal monitoring of the patients and results in substandard care.

“*So for the challenges, when we talk about the health workers, our numbers are just small. It doesn’t match up with the number of patients that we have on our wards, so sometimes we are not able to monitor them as we are supposed to.”* (Midwife, 33 years)

“*Lots of challenges especially with the staff strength. You see, a ward that has 50 beds and sometimes over because some patients are discharged and are not going home because they have to be on the floor, so we get more than that sometimes. And you have 3 midwives running such a ward, so the ratio you can imagine and then you have these hypertensive disorders in pregnancy, not just one, you have three, four, five.”* (Midwife, 30 years)

Most of the health professionals openly narrated instances where suboptimal monitoring of pregnant women with severe preeclampsia occurs due to inadequate number of health care staff and high patient load.

“*The most difficult aspects has to do with monitoring their BP on time. Yeah it doesn’t always happen on time. The drug administration too, the number of people too counts because when you start, by the time you get to the one with the high BP the time is far spent so they don’t get their medication too on time.”* (Midwife, 35 years)

#### Inappropriate attitudes of health providers

Some health providers indicated situations when delays in providing care for hypertensive mother may be due to the actions of the health providers themselves resulting in severe complications. There are unusual occurrences of misdiagnosis which results in delays in initiating appropriate treatment. In some situations, mistakes or professional errors (by omission or commission) on the part of the health professional result in avoidable complications and adverse outcomes. In the quote below, one medical officer mentions a practical scenario of misdiagnosis, delay in treatment initiation, and the potential for severe complications.

“*So if maybe you fail to diagnose a woman who is having BP on the high side and based on one or two reasons you forget ….. On the other hand if you diagnose but fail to institute treatment early or on time complications do set in.”* (Medical doctor, 31 years)

Some of the respondents described situations where some health workers absent themselves from work when they are expected to be at post. Similarly, issues related to inadequate information flow between the health workers were mentioned especially between the workers and their supervisors (inadequate inter-professional communication). These challenges usually lead to suboptimal provision of care and conflicts among the workers. Other attitudes related to standards of care include delays in timely provision of care due to the inactivity of the health workers as well as inadequate/irregular provision of relevant information to pregnant women.

*“We want to get surgeries done and the ‘orderly’ is not around; there are materials that are finished, you want people to call their in-charges to bring in the materials and they are just sitting there so in the end you have some conflicts.”* (Medical doctor, 40 years)

*“We the professionals, I think we should treat the women promptly; education should be constant and ongoing throughout the antenatal period. We don’t just say it at one visit and then stop. Every time we come into contact with these women we have to continue.”* (Medical doctor, 32 years)

## Discussion

In this qualitative study, we explored the real-life challenges experienced by health professionals in the clinical management of hypertensive disorders in pregnancy in health facilities in Ghana. The major challenges highlighted mainly relate to patient factors (inadequate understanding and misconceptions, financial burden and noncompliance with medical instructions), health systems factors (facility delays and lack of logistics, medications and laboratory support) and health provider factors including inadequate number of health professionals, inadequate patient education, inappropriate attitudes and professional errors.

Previous studies indicate women's suboptimal knowledge of preeclampsia partly accounts for the high incidence of complications and suboptimal outcomes (24, 25). In a study to assess women's knowledge on preeclampsia, only 43% of the questions were answered correctly by the women and the factors associated with higher awareness included literacy, previous history of preeclampsia, multiparity and prior health education on the condition (25). In this study, significant level of suboptimal knowledge on hypertension in pregnancy has been determined and this illustrates the necessity to optimally promote health literacy among pregnant women and their relations. In a recent study involving Moroccan pregnant women, approximately 50% had no knowledge about hypertension in pregnancy and its danger symptoms (24). Most of the women considered communicating information *via* movies as the most appropriate way to promote education on HDP (24).

In the typical clinical settings, some hypertensive women are unable to comply with the standard care prescribed by the health providers. Women's non-compliance may be due to the high level of misconception and traditional beliefs which constitute major challenges in providing high quality care and impact negatively on shared decision making. Spiritual inclinations coupled with misconceptions may also contribute significantly to unnecessary delays in treatment initiation for hypertensive mothers and may result in worse adverse outcomes (26). In this direction, promotion of maternal health literacy is crucial in improving care, creating awareness and optimizing pregnancy outcomes associated with maternal hypertension (24, 26). To achieve this, integration of maternal classes into antenatal care provides an important opportunity for promotion of the health education and this is associated with lowered risk of cesarean section and increased satisfaction with care (27).

In this study, health system challenges were considered a major hindrance to the provision of quality care for women with hypertension during pregnancy. In low resource settings, health systems do not usually function optimally due to the intrinsic bottlenecks and inappropriate policies. Compared with the population needs, there is evidence of significant deficiency in the health systems in SSA including health infrastructure, health expenditure and skilled health professionals resulting in substandard care (28). According to WHO, the health system consists of all organizations, people and actions whose primary intent is to promote, restore or maintain health. The WHO framework has six building blocks making up the health system comprising health services, health workforce, health information, medical technologies, health financing and leadership and governance (29). In this study nearly all the components of the health system's building blocks were mentioned as limitations in providing high quality of care for hypertensive pregnant women.

In contemporary management of hypertension in pregnancy, functional health systems are critical in reducing the associated severe morbidity and mortality. Institutional delays in provision of appropriate treatment accounts for significant complications and adverse pregnancy outcomes. Facility-based delays are partly attributed to staff shortage, lack of motivation, high workload, lack of beds or ward space, and financial limitation (30). For instance, financial burden on women remains a core obstacle to provision of high quality maternal care. The national health insurance scheme (NHIS) was introduced to eliminate the associated delays and achieve universal coverage. In this study, most of the health professionals indicated that the NHIS does not cover all the hospital bills for the hypertensive mothers. There was a general impression that the NHIS covers only part of the antihypertensive medications and laboratory tests which are generally costly. Most women are unable to afford the cost of treatment (medications and laboratory tests) making clinical decision-taking challenging. In a recent study in Ghana, Vestering et al. describes how health providers innovatively navigate the logic of care for women with hypertension in pregnancy given the prevailing health system and patient related challenges (31). In this situation, poverty eradication is requisite for sustainable development and remains the greatest global challenge worldwide especially in LMICs (1, 32). There is the need for urgent revamp in the health systems to optimize the available scarce resources to improve the quality of maternal care.

Similarly, logistics related challenges including frequent breakdown or shortage of blood pressure measuring tools are a core contributor to suboptimal care in LMICs (30, 33–35). These limitations are attributed to frequent use and high turnover rate due to large numbers of hypertensive mothers. The frequent shortage of the blood pressure measuring instruments precludes optimal monitoring of women with preeclampsia with a potential for poor pregnancy outcomes. In a recent related survey in South Africa, more than half of the practicing midwives indicated frequent shortage of basic essential equipment such as BP measuring machines (35) similar to the current findings. Similarly, frequent unavailability of essential medications (antihypertensives) and blood products remains a major challenge in the management of HDP in pregnancy especially in LMICs (34). Unavailability of safe blood products for transfusion accounts for a significant proportion of maternal deaths especially in low resource settings (30, 36). Furthermore, challenges associated with laboratory tests for hypertensive women is paramount and affect clinical decision especially in emergency cases.

Another important health system challenge was the recurrent shortage of beds for admitting the pregnant women, frequently described as the “no bed syndrome”. This normally occurs when the health facility is overwhelmed with cases and all the beds are occupied. In most cases, pregnant women are referred from a lower-level facility to the larger hospitals and major complications might occur in the process. Relatedly, the issue of inadequate number of health professionals and poor professional attitudes negatively impacts the provision of the optimal quality of care for hypertensive mothers. Frequent shortages of health staff is endemic in LMICs and partly accounts for the severe maternal morbidity and mortality (30, 35). In a similar study in Zimbabwe, suboptimal monitoring of hypertensive mother was partly attributed to frequent shortage of human resources (33) as also reported in this study.

In minimizing patient-related challenges, strengthening family support system emerged prominently from almost all the categories of health professionals. Clinical management of women with hypertension in pregnancy is multi-dimensional and the role of the family members is indispensable in achieving success and preventing major complications. Majority of the doctors and midwives recounted that the family members including the husbands/partners have poor knowledge of or are completely unaware about the conditions the women are suffering from. Adequate health education promotion for the immediate family members will improve their understanding and facilitate co-operation and social support. Similarly, regular provision of health education to women during pregnancy and the entire population (*via* film, social media and demonstrations including role play), addressing shortage of staff and inappropriate professional attitude, promotion of blood donations, and expanding NHIS coverage (admittance, medication) constitute viable entry points in optimizing clinical care for hypertensive pregnant women in the country.

In addition, provision of essential medications, blood pressure apparatuses and other basic logistics, and human resource are considered primary responsibilities of the government to ensure improved quality of maternal care and universal health coverage. In the end, political commitment remains the cornerstone in optimizing the clinical management of HDP especially in LMICs.

### Strengths and limitations

The key strengths of the study include the use of in-depth interviews (IDIs) and multicenter design to explore the experiences of health workers concerning the challenges in managing maternal hypertension. IDI is considered one of the most powerful approaches for gaining significant understanding and exploring specific concepts and topics into details because as it allows for spontaneity, flexibility, and responsiveness to the respondents (23). The main limitation of our study centers on non-inclusion of focused group discussion (FGD) which would have generated a comprehensive discussion of the subject and emergence of relevant key themes. FGD generates a dynamic and interactive discussions among participants resulting in the emergence of multiple stories and display of diverse experiences. Another limitation relates to the use of one interviewer for all the IDIs. While a single interviewer results in more efficiency in the data collection the use of more interviewers (with different reflexivity strengths) provides multiple observations and different perspectives as well as confirmation of the findings from different sources (investigator triangulation) (23). Despite the enlisted limitations, this study highlights relevant insights into context-specific challenges associated with clinical management of maternal hypertension. The findings of this study may be transported to other LMICs with similar settings.

## Conclusion

This study explored major challenges observed by health professionals associated with the clinical management of hypertension in pregnancy. These challenges include patient-related factors (including inadequate awareness, non-compliance with medical instructions, financial limitations and misconceptions), health system-related challenges (including frequent unavailability of logistics and medications) and health provider-associated issues (inadequate number of health staff and poor attitudes). The findings of this study underscore the importance of an integrative approach to tackling health system challenges. Context-specific interventions such as regular health education promotion, re-structuring of the health systems through improvement in health insurance coverage and government subsidy for hypertensive mothers are recommended to inform policy change and improve the quality of clinical care for women with hypertension in pregnancy.

## Data Availability

The raw data supporting the conclusions of this article will be made available by the authors, without undue reservation.
